# Cases of Deformity from Burns, Successfully Treated by Plastic Operations

**Published:** 1844-10

**Authors:** 


ART. I.—*Cases of Deformity from Burns, successfully treated by Plastic Operations.* By THOMAS D. MÜTTER, M.D., Professor of Surgery in Jefferson Medical College, Philadelphia, &c.—*Philadelphia*, 1843. 8vo, pp. 24.

THE present, together with two other pamphlets, treating of closely allied branches of the healing art by the same author, have just reached us; and we are not willing to pass by the present opportunity of glancing at the performance of Dr. Mütter, although we have the intention of giving the subject of Plastic Surgery, as brought to bear on "the human face divine," a more critical notice and ampler space in an early Number of our Journal. There are few, if any, deformities consequent on accident, short of the irreparable loss of limbs, which have so successfully set at defiance the *art* of surgery for their removal, as those occasioned by burns; indeed the surgeon has felt that, in the production of the deformity which he could fully anticipate and had ample leisure to watch, nature's antagonising powers have overmatched him. We have painfully proved this in a case recently under our own care, in which a large surface of the skin was destroyed, involving the axilla, upper arm, and side. To meet the anticipated contraction, we had an apparatus constructed by a clever mechanist, with a lengthening screw connected to a pad adapted to the side and to a support for the arm; but we were much disappointed by the occurrence of an evil greater than that we sought to obviate, viz. a commencing curvature of the spine (the patient being a boy), resulting from the constrained position assumed by the child, to relieve the distress consequent on the stretching of the granulating surface. We, therefore, hail with satisfaction any attempt to remedy evils which have been either from neglect or unavoidably produced; and we cordially congratulate Dr. Mütter on the success which seems to have attended his operations. We shall satisfy ourselves (for the reason above stated) with merely noticing, for the present, the *result* of Dr. Mütter's treatment in these cases; the *modus operandi* and other details we shall leave for another opportunity.

The first case of a severe burn, involving the face, throat, and upper part of the thorax, and which occurred during childhood, is thus graphically described, as to its effects, by the young lady who was the subject of it: "I have been unable to throw my head to the left side or backwards, or to close my mouth for more than a few seconds at a time for twenty-three years. My right eye was also drawn down some distance below the other, and when I endeavoured to turn my head, it was entirely closed. My condition has been most humiliating and made life a burden; but having good health, I strove to reconcile myself to my hard lot."

We also give the result and consequences of the operation in the patient's words: "The comfort and satisfaction I feel cannot be expressed; your exertions in my behalf have been blessed far beyond my most sanguine expectations. You have set my head at liberty, so that I can turn it any way at pleasure and without pain; you have relieved the drawings of my eye; and I am also enabled to close my mouth with comfort, a blessing that cannot be described." The accompanying figures amply confirm the accounts, and exhibit a remarkable contrast in the appearance of the patient. The second and third cases detailed were of the same nature, and the results marked by the same benefit and improvement. Some excellent "remarks are added," involving the consideration of "the nature of the tissue to be divided or removed;" "the thickness or profundity of the cicatrix, its location, extent, and age;" and lastly, on the "peculiar deformity of the cicatrix," whether narrow, prominent, or complicated by extensive adhesions.

We shall have great pleasure in resuming a more intimate acquaintance with Dr. Mütter's present productions; and shall be pleased, as we have reason to expect, if they lead to an introduction to some more comprehensive work, by which we may judge of the present state of transatlantic surgery.

